# Long-term safety of Ixekizumab in adults with psoriasis, psoriatic arthritis, or axial spondyloarthritis: a post-hoc analysis of final safety data from 25 randomized clinical trials

**DOI:** 10.1186/s13075-023-03257-7

**Published:** 2024-02-12

**Authors:** Atul Deodhar, Andrew Blauvelt, Mark Lebwohl, Meghan Feely, Andris Kronbergs, Nadezhda Eberhart, Danting Zhu, Elsa Inman, Elsie Grace, Thorsten Holzkaemper, Proton Rahman, Helena Marzo-Ortega, Kim A. Papp, Joseph F. Merola, Alice B. Gottlieb, Sergio Schwartzman

**Affiliations:** 1 Oregon Health & Science University, Portland, OR USA; 2 Oregon Medical Research Center, Portland, OR USA; 3 Mount Sinai Hospital, New York, NY USA; 4 Eli Lilly and Company, Indianapolis, IN USA; 5 Mount Sinai West and Mount Sinai Morningside, New York, NY USA; 6 Memorial University of Newfoundland, St. John’s, NL Canada; 7 NIHR Leeds Biomedical Research Centre, Leeds Teaching Hospitals Trust, Leeds Institute for Rheumatic and Musculoskeletal Medicine, The University of Leeds, Leeds, UK; 8 Probity Medical Research and Alliance Clinical Trials, Waterloo, ON Canada; 9 Division of Dermatology, Department of Medicine, University of Toronto, Toronto, ON Canada; 10 The University of Texas Southwestern Medical Center, Dallas, TX USA; 11 Department of Dermatology, Icahn School of Medicine at Mount Sinai, New York, NY USA; 12 72Nd Street Medical Associates, Scarsdale, NY USA

**Keywords:** Ixekizumab, Psoriasis, Psoriatic arthritis, Axial Spondyloarthritis, Long-term Safety, Integrated Safety

## Abstract

**Background:**

We report long-term, end-of-study program safety outcomes from 25 randomized clinical trials (RCTs) in adult patients with psoriasis (PsO), psoriatic arthritis (PsA), or axial spondyloarthritis (axSpA) [including ankylosing spondylitis (AS) and non-radiographic axial spondyloarthritis (nr-axSpA)] who received ≥ 1 dose of Ixekizumab (IXE) over 5 years (PsO) or up to 3 years (PsA, axSpA).

**Methods:**

This integrated safety analysis consists of data from patients who received any dose of IXE, across 25 RCTs (17 PsO, 4 PsA, 4 axSpA). Rates of treatment-emergent adverse events (TEAEs), serious adverse events (SAEs) and selected adverse events (AEs) of interest were analyzed for all pooled studies by years of therapy and overall, through March 2022. Results were reported as exposure-adjusted incidence rates (IRs) per 100 patient-years (PY) overall and at successive year intervals.

**Results:**

Six thousand eight hundred ninety two adult patients with PsO, 1401 with PsA, and 932 with axSpA (including AS and nr-axSpA), with a cumulative IXE exposure of 22,371.1 PY were included. The most commonly reported TEAE across indications was nasopharyngitis (IRs per 100 PY: 8.8 (PsO), 9.0 (PsA), 8.4 (axSpA)). SAEs were reported by 969 patients with PsO (IR 5.4), 134 patients with PsA (IR 6.0), and 101 patients with axSpA (IR 4.8). Forty-five deaths were reported (PsO, *n* = 36, IR 0.2; PsA, *n* = 6, IR 0.3; axSpA, *n* = 3, IR 0.1). TEAEs did not increase during IXE exposure: IRs per 100 PY, PsO: 88.9 to 63.2 (year 0–1 to 4–5), PsA: 87 to 67.3 (year 0–1 to 2–3), axSpA: 82.1 to 55.4 (year 0–1 to >  = 2). IRs per 100 PY of discontinuation from IXE due to AE were 2.9 (PsO), 5.1 (PsA), and 3.1 (axSpA). IRs per 100 PY of injection site reactions were 5.9 (PsO), 11.6 (PsA) and 7.4 (axSpA); *Candida*: 1.9 (PsO), 2.0 (PsA), and 1.2 (axSpA); depression, major adverse cerebro-cardiovascular events and malignancies: ≤ 1.6 across all indications. Adjudicated IRs per 100 PY of inflammatory bowel disease were ≤ 0.8 across indications (0.1 [PsO]; 0.1 [PsA]; 0.8 [axSpA]).

**Conclusions:**

In this integrated safety analysis, consisting of over 22,000 PY of exposure, the long-term safety profile of IXE was found to be consistent with previous, earlier reports, with no new safety signals identified.

**Trial registration:**

NCT registration numbers for RCTs included in this integrated analysis can be found in Additional File 1.

**Supplementary Information:**

The online version contains supplementary material available at 10.1186/s13075-023-03257-7.

## Background

Psoriasis (PsO), psoriatic arthritis (PsA), and axial spondyloarthritis (axSpA) (including ankylosing spondylitis [AS] and non-radiographic axSpA [nr-axSpA] are chronic immune-mediated inflammatory diseases associated with cardiovascular and metabolic comorbidities [1–3]. Biologic therapies are now widely recognized as effective treatment options for these immune-mediated inflammatory conditions. Because of the immunomodulatory effects of biologic medications, the potential for adverse events (AEs), the prolonged treatment periods typically required to manage these immune-mediated diseases, and the increased burden of comorbidities experienced by patients, continued safety monitoring is essential.

Interleukin (IL)-17A is an inflammatory cytokine and a key therapeutic target for PsO, PsA, and axSpA, playing a pivotal role in the pathogenesis of these immune-mediated diseases [4–7]. Ixekizumab (IXE), a high-affinity monoclonal antibody that selectively targets IL-17A [8], is approved for the treatment of moderate-to-severe plaque PsO, PsA, AS, nr-axSpA, and pediatric PsO [8–11]. Since long-term use of IXE is required to control disease across these indications, accurate and timely reporting of safety data, commensurate with increasing patient-years (PY) of exposure is essential to ongoing safety monitoring in these patients. Long-term safety of IXE was reported in a previous integrated analysis study [12]. Since this previous analysis, the last long-term exposure study has concluded. Here, we present the final, end-of-study program update, examining integrated safety data, following long-term IXE treatment in adult patients with PsO (up to 6 years exposure), PsA or axSpA (including patients with AS and nr-axSpA) (up to 3 years).

## Methods

### Patients and studies

Patient data were integrated from 25 randomized, controlled clinical trials examining IXE treatment among adult patients with PsO (3 Phase 1, 1 Phase 2, 11 Phase 3, 2 Phase 4; *N* = 6,892), PsA (3 Phase 3, 1 Phase 3b/4; *N* = 1401), and axSpA (4 Phase 3; *N* = 932, including AS and nr-axSpA). Study designs and eligibility criteria for these studies were described elsewhere [12–18]. Table S1 outlines the baseline risk of trial participants relevant to safety in the current study.

Briefly, for the PsO studies included in this analysis, pooled safety data for patients who were candidates for phototherapy and/or systemic therapy and met the following criteria were included in the analyses: aged ≥ 18 years with moderate-to-severe plaque PsO (≥ 10% body surface area involvement, Static Physician’s Global Assessment of ≥ 3, Psoriasis Area and Severity Index ≥ 12 at baseline). UNCOVER-1, -2, and -3 were the largest trials for this patient cohort and contributed the most patient safety data for analysis. Studies UNCOVER-1, -2 and -3 had 12-week, randomized, placebo-controlled periods. UNCOVER-2 and -3 additionally had an etanercept group up to week 12 [8, 19]. The total study treatment period (not including the entry period) for trial I1F-MC-RHAJ(d), in which the safety (among other outcomes) of IXE subcutaneous dosing groups was assessed (compared to placebo), in adults with plaque psoriasis was 364 weeks (protocol on file at Eli Lilly and Company).

Analyses were performed on pooled safety data relating to patients in the PsA cohort who participated in trials, SPIRIT-P1, -P2, -P3, and SPIRIT H2H. SPIRIT-P1 and -P2 were phase 3, double-blinded, randomized, parallel-group, placebo (PBO)-controlled trials with similar study designs [15, 20–22]. SPIRIT-P1 patients were biologic-naïve and could not have been taking more than one conventional synthetic disease-modifying anti-rheumatic drug (csDMARD) or more than four csDMARDs before enrollment, while SPIRIT-P2 patients were csDMARDs and biologic (b) DMARDs experienced. SPIRIT-P1 additionally included assessments of radiographic progression and used adalimumab as an active control to week 24. SPIRIT-P3 was a phase 3 study consisting of an open-label period, followed by a randomized double-blinded withdrawal period, which examined IXE treatment in patients with active PsA, who were csDMARD-inadequate responders and bDMARD-naive. SPIRIT-P1, -P2 and P3 assessed the safety of IXE treatment up to 156 weeks. SPIRIT-H2H was an open-label, randomized, multicentre, assessor-blinded, parallel-group study that evaluated the efficacy and safety of IXE versus adalimumab and concomitant MTX in biologic-naïve, csDMARD-inadequate-responder patients with active PsA and PsO.

For the axSpA cohort, pooled safety data were integrated from the COAST-V (biologic-naïve) [23], COAST -W (tumour necrosis factor (TNF) inhibitor-experienced) [24], COAST -X (biologic naïve) [25], and COAST -Y trials [26]. Patients who participated in the COAST-V and -W trials presented with radiographic axSpA (r-axSpA), thus fulfilling both the Assessment of Spondyloarthritis International Society (ASAS) and modified NY criteria based on the presence of sacroiliitis on X-rays. Patients who participated in the COAST-X trial were classified as nr-axSpA patients, who fulfilled ASAS criteria and had a treatment history for axSpA for at least 12 weeks. In these studies, patients were randomized to placebo, adalimumab (active reference arm, COAST-V only) or IXE. COAST-Y was the 2-year extension study that enrolled patients who successfully completed COAST-V, COAST-W and COAST-X studies. Study designs of COAST-V, COAST-W, COAST-X and COAST-Y are described elsewhere [23–27]. Safety data were analysed in all patients having received ≥ 1 IXE dose.

### Safety outcomes and assessments

Safety analyses included pooled data from all patients who had received ≥ 1 dose of IXE. Results presented in the current study incorporate pooled data from all doses of IXE used in patients across the 25 RCTs under investigation, regardless of rescue medication or dose change. Data were pooled at the individual patient level for all IXE treatment groups, separately for the studies under investigation, by indication, and analyzed from the date of treatment initiation up to 31 March 2022. AEs were classified according to the Medical Dictionary for Regulatory Activities (MedDRA) (version 24.1 for PsO and axSpA-related studies, version 23.1 for PsA-related studies). A treatment-emergent adverse event (TEAE) was defined as an event that first occurred or worsened in severity after baseline and on or before the last day of the treatment period. Selected AEs of interest included infections, *Candida* infection, injection site reactions (ISRs), depression and suicide/self-injury, latent tuberculosis (TB), major adverse cerebro-cardiovascular events (MACE), malignancies, inflammatory bowel disease (IBD) and uveitis. Potential and suspected cases of MACE and IBD in Phase 3 trials were externally adjudicated. MACE events were defined as vascular death, non-fatal myocardial infarction and non-fatal stroke. Depression was measured using Quick Inventory of Depressive Symptomatology – Self Report 16 items. Selected AEs of interest (serious or non-serious) were defined as events of scientific and medical concern specific to the sponsor’s product or program. Serious AEs (SAEs) were any event meeting the International Conference on Harmonisation E2A seriousness criteria. Latent TB was defined either by latent TB preferred term (PT) or a positive result on any of the following annual tests: interferon-gamma release assay, mycobacterium TB complex test, or a tuberculin skin test. Risks for AEs, SAEs, and selected AEs of interest were expressed as exposure-adjusted incidence rates (EAIRs) per 100 PY for the treatment period under examination.

### Statistical analysis

Analyses were conducted on data from patients who received ≥ 1 dose of IXE, with data pooled for treatment groups by indication. An analysis across the IXE treatment period was performed on safety data pooled at the patient level, by indication and includes data from the beginning of the studies to March 2022 (PsO and axSpA cohorts) and March 2021 (PsA cohort). Overall exposure was described in PY and calculated as the sum of duration of IXE exposure (days) for all patients in the treatment group, divided by 365.25. AEs, SAEs, and selected AEs of interest were expressed as exposure-adjusted incidence rates (EAIRs) per 100 PY for the entire treatment period (i.e., calculated by dividing the number of patients with at least 1 event by the amount of accumulated person time, multiplied by 100) and by yearly intervals. In the yearly interval analysis, only person time accrued during the interval is considered. The same patient can be counted in multiple intervals as long as the AE of interest is observed in the corresponding interval. A likelihood ratio test of treatment effect from the Poisson regression model was used to calculate confidence intervals for IRs. Only descriptive statistics will be reported for safety analysis and baseline characteristics without statistical comparisons. Statistical analyses were performed using SAS® software version 9.4 or higher (SAS Institute).

## Results

### Patients

In this final analysis, a total of 6,892 patients with PsO, 1,401 patients with PsA and 932 patients with axSpA (including patients with AS and nr-axSpA) received > 1 dose of IXE and were included in this analysis, with a cumulative IXE exposure of 22,371.1 PY (18,025.7 PY for PsO, 2,247.7 PY for PsA, and 2,097.7 PY for axSpA) (representing an extended IXE exposure period of 1,475.2 PY from the previous report [12]). Patient data were pooled from 25 clinical trials, by indication. The maximum exposure was 2,236 days for patients with PsO (owing to the length of study time associated with trial I1F-MC-RHAJ(d)), 1,219 days for patients with PsA, and 1,241 days for patients with axSpA (Figure S1). The median duration of exposure was 478.5 days (1.3 years) for patients with PsO, 504.5 days (1.4 years) for patients with PsA, and 981.0 days (2.7 years) for patients with axSpA.

### Demographic and baseline characteristics

Demographics and baseline characteristics for the overall patient population are presented in Table 1. Of note, the axSpA cohort includes patients with AS and nr-axSpA. The mean (standard deviation, SD) age of patients in the PsO cohort was 45.7 (13.2) years, 49.1 (11.9) in the PsA cohort and 42.8 (12.6) in the axSpA cohort. Across all 3 indications, the mean age was 45.9 years. The majority of patients in the PsO and axSpA cohorts were male (68.1% and 69.7%, respectively), while the proportion of male to female patients in the PsA cohort was largely balanced (48.5% vs. 51.5%, respectively). Over 70% of participants across indications were white. The mean body mass index (BMI) of patients with PsO was comparable to those with PsA (30.4 kg/m^2^ vs. 30.0 kg/m^2^), and slightly higher than patients with axSpA (27.5 kg/m^2^). In the PsO cohort, 35.9% of patients were naïve to systemic therapy, 35.1% previously received non-biologic therapy, 12.0% received biologic therapy, and 17.0% received both biologic and non-biologic therapy. The proportion of patients who were naïve to systemic therapy was lower among patients with PsA (20.7%). In the PsA cohort, 55.2% of patients previously received non-biologic therapy, 5.1% received biologic therapy, and 19.1% received both biologic and non-biologic therapy. Just 0.6% of patients with axSpA were naïve to systemic therapy, having never received non-steroidal anti-inflammatory drugs (NSAIDs), csDMARDs or bDMARDs. 99.2% of those who previously underwent therapy received non-biologic therapy, 32.7% received biologic therapy, and 32.6% received both biologic and non-biologic therapy. Patients undergoing concomitant therapy with NSAIDs, csDMARDs or oral corticosteroids (CSs) were included in this analysis. The lowest proportion of patients who used oral CSs were those with PsO (6.1%), the highest was among patients with PsA (25.5%), while 21.9% of patients with axSpA demonstrated use of oral CSs. The proportion of patients previously/currently undergoing concomitant therapy with NSAIDs was highest among those with axSpA (91.3%) compared with patients with PsO (41.2%) or PsA (65.5%). More patients with PsA (71.5%) demonstrated concomitant use of csDMARDs than patients with PsO (11.4%) or axSpA (40.6%).

**Table 1:** Demographic and baseline characteristics for the overall patient population

**Characteristics**	**Integrated PsO IXE (*N* = 6892)**	**Integrated PsA IXE (*N* = 1401)**	**Integrated axSpA IXE**^b^** (*N* = 932)**
Age, years, mean (S.D.)	45.7 (13.2)	49.1 (11.9)	42.8 (12.6)
Sex, n (%)			
Male	4696 (68.1)	679 (48.5)	650 (69.7)
Female	2196 (31.9)	722 (51.5)	282 (30.3)
Race, n (%)			
White	5612 (81.5)	1278 (91.3)	689 (74.1)
Black/African American	206 (3.0)	4 (0.3)	4 (0.4)
Hispanic/Latino	786 (12.0)	130 (9.3)	209 (22.4)
Asian	914 (13.3)	68 (4.9)	181 (19.5)
Geographic region, n (%)			
North America	3595 (52.2)	293 (20.9)	107 (11.5)
Europe	2251 (32.7)	896 (64.0)	462 (49.6)
Asia	244 (3.5)	62 (4.4)	170 (18.2)
Weight, kg, mean (SD)	90.3 (23.5)	86.1 (20.3)	80.0 (17.3)
BMI, kg/m2, mean (S.D.)	30.4 (7.3)	30.0 (6.9)	27.5 (5.7)
Tobacco use (current), n (%)	874 (12.7)	538 (38.4)	276 (29.6)
Alcohol consumption (yes), n (%)	1421 (20.6)	718 (51.4)	399 (42.8)
COPD, n (%)	180 (2.6)	43 (3.1)	22 (2.4)
Asthma, n (%)	377 (5.5)	88 (6.3)	38 (4.1)
Diabetes, n (%)	751 (10.9)	169 (12.1)	50 (5.4)
Inflammatory bowel disease, n (%)	44 (0.6)	14 (1.0)	30 (3.2)
Duration of symptoms in years, mean (S.D.)	18.7 (12.2)	9.4 (8.6)	15.2 (10.9)
Previous medical history, n (%)			
Herpes zoster	51 (0.7)	19 (1.4)	14 (1.5)
*Candida* infections	16 (0.2)	4 (0.3)	0
Latent tuberculosis infections	118 (1.7)	35 (2.5)	76 (8.2)
Psychiatric disorders	78 (1.1)	18 (1.3)	12 (1.3)
Previous systemic therapy, n (%)^a^			
Never used	2474 (35.9)	290 (20.7)	6 (0.6)
Non-biologic	2418 (35.1)	773 (55.2)	925 (99.2)
Biologic	826 (12.0)	71 (5.1)	305 (32.7)
Biologic and non-biologic	1174 (17.0)	267 (19.1)	304 (32.6)
Concomitant therapy, n (%)			
NSAIDs	2838 (41.2)	917 (65.5)	851 (91.3)
csDMARDs	783 (11.4)	1002 (71.5)	378 (40.6)
Oral CSs	422 (6.1)	357 (25.5)	204 (21.9)

*Abbreviations: AS* Ankylosing spondylitis, *axSpA* Axial spondyloarthritis, *BMI* Body mass index, *CSs* Corticosteroids, *csDMARDs* Conventional synthetic disease-modifying anti-rheumatic drugs, *COPD* Chronic obstructive pulmonary disease, *IXE* Ixekizumab, *n* Number of patients per category, *N* Number of patients in the analysis population, *nr-axSpA* Non-radiographic axial spondyloarthritis, *NSAIDs* Non-steroidal anti-inflammatory drugs, *PsA* psoriatic arthritis, *PsO* Psoriasis, *SD* Standard deviation

‘Integrated’ refers to data obtained from all eligible patients who received any dose of IXE

^a^systemic therapy in patients with PsO and PsA are either non-biologic only, biologic only, or biologic and nonbiologic. Patients with axSpA could have both previous therapies

^b^Includes patients with AS and nr-axSpA

### Overall safety summary

#### TEAEs

**Fig. 1:**
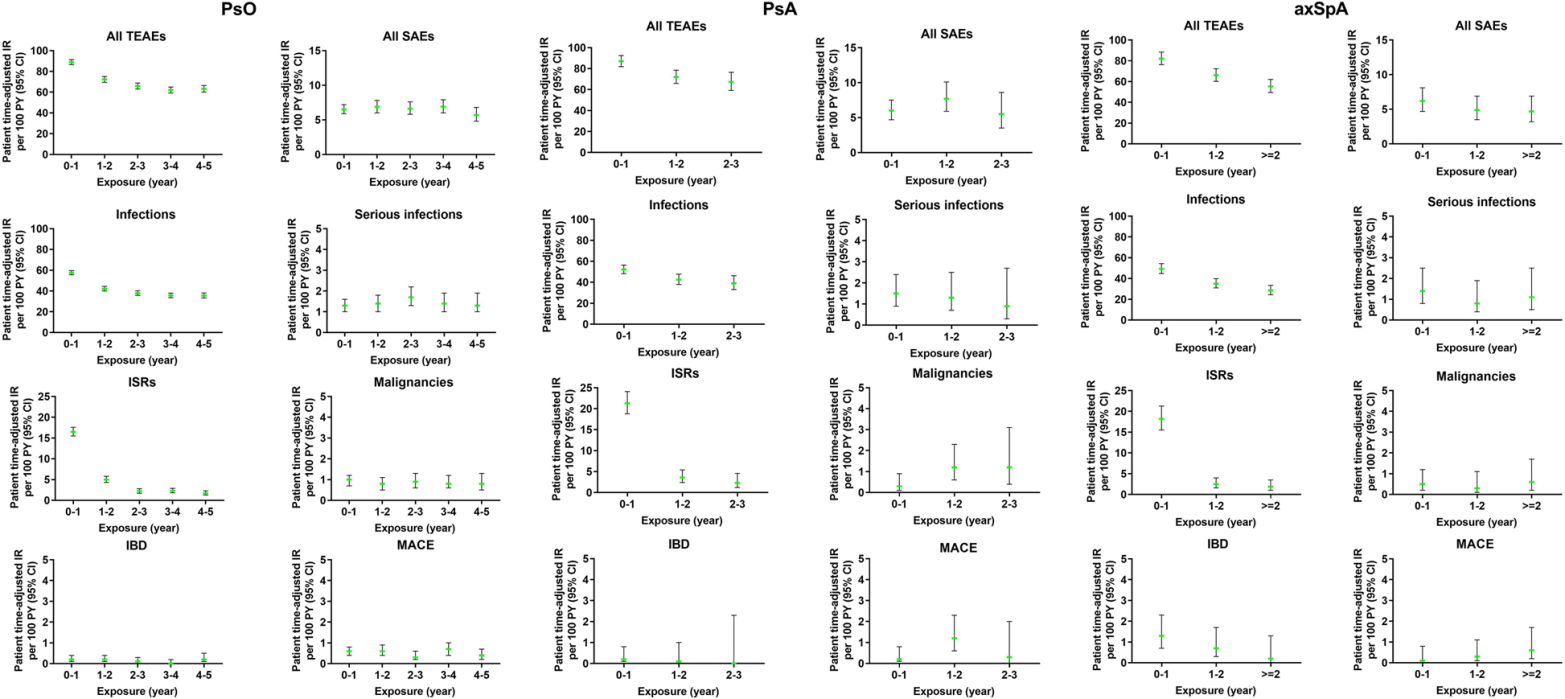
Exposure-adjusted IRs of TEAEs, SAEs, and selected AEs (Exposure Safety Populations) across indications over successive year intervals; PsO (year 0 to year 5) PsA (year 0 to year 3), axSpA (including patients with AS and nr-axSpA) (year 0 to ≥ 2). The data points on the graph are the IR (95% CI)/100 PY. The CIs for the IRs are from likelihood ratio test of treatment effect from the Poisson regression model. All studies were conducted using the original, citrate-containing formulation of IXE. Abbreviations: axSpA, axial spondyloarthritis; IBD, inflammatory bowel disease; CI; confidence interval; IR, incidence rate per 100 patient-years; ISR, injection site reaction IXE; ixekizumab; MACE, major adverse cerebro-cardiovascular event; N, number of patients in the analysis population; n, number of patients in each category; PsA, psoriatic arthritis; PsO, psoriasis; SAE, serious adverse event; TEAE, treatment-emergent adverse event

The IRs per 100 PY for any TEAE were 32.5 for patients with PsO, 50.3 for patients with PsA, and 38.0 for patients with axSpA, representing 85% (*n* = 5857) of patients with PsO, 80.7% (*n* = 1131) of patients with PsA, and 85.6% (*n* = 798) of patients with axSpA. The majority of TEAEs experienced by patients across indications were mild or moderate in severity (Table 2). Severe TEAEs were reported by 15.0% of patients with PsO (incidence rate [IR] 5.7 per 100 PY), 8.1% of patients with PsA (IR 5.1 per 100 PY), and 11.1% of patients with axSpA (IR 4.9 per 100 PY). TEAEs reported across the studies decreased over the observational periods examined (Fig. 1). The most frequently reported TEAEs (≥ 10%) across indications were nasopharyngitis (23.1% PsO, 14.4% PsA, 18.9% axSpA) and ISR (10.1% PsO, 11.1% PsA, 10.0% axSpA) (Table 2). IRs of nasopharyngitis ranged from 8.4 to 9.0 per 100 PY, and IRs of ISR ranged from 3.9 to 6.9 per 100 PY. Four cases of COVID-19 were reported among patients with axSpA who participated in the COAST-Y trial [27] (0.4%, IR 0.2 per 100 PY) (COAST-Y was the only clinical trial under investigation still active during the pandemic). Three of these cases were mild (0.3%), IR 0.1 per 100 PY), 1 case was moderate in severity (0.1%, IR 0.0 per 100 PY).

**Table 2:** Pooled safety outcomes across indications

	**Pooled PsO IXE (*N* = 6892)**			**Pooled PsA IXE (*N* = 1401)**			**Pooled axSpA IXE (*N* = 932)**		
Total patient-years	18025.7			2247.7			2097.7		
Maximum exposure (days)	2236			1219			1241		
	**n (%)**	**IR**	**95% CI of IR**	**n (%)**	**IR**	**95% CI of IR**	**n (%)**	**IR**	**95% CI of IR**
**TEAEs**^a,b^	5857 (85.0)	32.5	31.7, 33.3	1131 (80.7)	50.3	47.5, 53.3	798 (85.6)	38.0	35.5, 40.8
Mild	1799 (26.1)	10.0	9.5, 10.5	461 (32.9)	20.5	18.7, 22.5	276 (29.6)	13.2	11.7, 14.8
Moderate	3025 (43.9)	16.8	16.2, 17.4	556 (39.7)	24.7	22.8, 26.9	419 (45.0)	20.0	18.2, 22.0
Severe	1032 (15.0)	5.7	5.4, 6.1	114 (8.1)	5.1	4.2, 6.1	103 (11.1)	4.9	4.0, 6.0
**Most Common TEAEs**^n^									
Nasopharyngitis	1592 (23.1)	8.8	8.4, 9.3	202 (14.4)	9.0	7.8, 10.3	176 (18.9)	8.4	7.2, 9.7
Upper respiratory tract infection	1114 (16.2)	6.2	5.8, 6.6	186 (13.3)	8.3	7.2, 9.6	122 (13.1)	5.8	4.9, 6.9
Injection site reaction	698 (10.1)	3.9	3.6, 4.2	156 (11.1)	6.9	5.9, 8.1	93 (10.0)	4.4	3.6, 5.4
Arthralgia	642 (9.3)	3.6	3.3, 3.8	34 (2.4)	1.5	1.1, 2,1	66 (7.1)	3.1	2.5, 4.0
Headache	541 (7.8)	3.0	2.8, 3.3	56 (4.0)	2.5	1.9, 3.2	41 (4.4)	2.0	1.4, 2.7
Back pain	447 (6.5)	2.5	2.3, 2.7	65 (4.6)	2.9	2.3, 3.7	50 (5.4)	2.4	1.8, 3.1
Hypertension	433 (6.3)	2.4	2.2, 2.6	64 (4.6)	2.8	2.2, 3.6	46 (4.9)	2.2	1.6, 2.9
Bronchitis	410 (5.9)	2.3	2.1, 2.5	91 (6.5)	4.0	3.3, 5.0	72 (7.7)	3.4	2.7, 4.3
Diarrhoea	387 (5.6)	2.1	1.9, 2.4	61 (4.4)	2.7	2.1, 3.5	60 (6.4)	2.9	2.2, 3.7
Sinusitis	384 (5.6) 364	2.1	1.9, 2.4	77 (5.5)	3.4	2.7, 4.3	39 (4.2)	1.9	1.4, 2.5
Urinary Tract Infection	(5.3)	2.0	1.8, 2.2	69 (4.9)	3.1	2.4, 3.9	45 (4.8)	2.1	1.6, 2.9
Pharyngitis	307 (4.5)	1.7	1.5, 1.9	54 (3.9)	2.4	1.8, 3.1	61 (6.5)	2.9	2.3, 3.7
Injection site erythema	203 (2.9)	1.1	1.0, 1.3	60 (4.3)	2.7	2.1, 3.4	33 (3.5)	1.6	1.1, 2.2
Cough	334 (4.8)	1.9	1.7, 2.1	48 (3.4)	2.1	1.6, 2.8	17 (1.8)	0.8	0.5, 1.3
**SAEs**^o^	969 (14.1)	5.4	5.0, 5.7	134 (9.6)	6.0	5.0, 7.1	101 (10.8)	4.8	4.0, 5.9
**Deaths**	36 (0.5)	0.2	0.1, 0.3	6 (0.4)	0.3	0.1, 0.6	3 (0.3)	0.1	0.0, 0.4
**AE leading to discontinuation (including death)**	519 (7.5)	2.9	2.6, 3.1	115 (8.2)	5.1	4.3, 6.1	66 (7.1)	3.1	2.5, 4.0
**Selected AEs of interest**									
Infections	4307 (62.5)	23.9	23.2, 24.6	759 (54.2)	33.8	31.4, 36.3	540 (57.9)	25.7	23.7, 28.0
Serious Infections	231 (3.4)	1.3	1.1, 1.5	28 (2.0)	1.2	0.9, 1.8	23 (2.5)	1.1	0.7, 1.6
* Opportunistic Infections*	536 (7.8)	3.0	2.7, 3.2	86 (6.1)	3.8	3.1, 4.7	28 (3.0)	1.3	0.9, 1.9
Oral candidiasis	144 (2.1)^c^	0.8	0.7, 0.9	16 (1.1)^d^	0.7	0.4, 1.2	5 (0.5)^e^	0.2	0.1, 0.6
Oral fungal infection^f^	11 (0.2)	0.1	0.0, 0.1	6 (0.4)	0.3	0.1, 0.6	3 (0.3)	0.1	0.0, 0.4
Esophageal candidiasis	14 (0.2)	0.1	0.0, 0.1	2 (0.1)	0.1	0.0, 0.4	4 (0.4)	0.2	0.1, 0.5
Herpes zoster	120 (1.7)	0.7	0.6, 0.8	16 (1.1)	0.7	0.4, 1.2	12 (1.3)	0.6	0.3, 1.0
* Candida Infections*	337 (4.9)	1.9	1.7, 2.1	45 (3.2)	2.0	1.5, 2.7	26 (2.8)	1.2	0.8, 1.8
Oral *Candida*^g^	160 (2.3)	0.9	0.8, 1.0	22 (1.6)	1.0	0.6, 1.5	8 (0.9)	0.4	0.2, 0.8
Vulvovaginal *Candida*^h^	97 (4.4)	1.7	1.4, 2.1	13 (1.8)	1.1	0.7, 2.0	7 (2.5)	1.2	0.6, 2.5
Skin *Candida*	52 (0.8)	0.3	0.2, 0.4	5 (0.4)	0.2	0.1, 0.5	2 (0.2)	0.1	0.0, 0.4
Esophageal candidiasis	16 (0.2)	0.1	0.1, 0.1	2 (0.1)	0.1	0.0, 0.4	5 (0.5)	0.2	0.1, 0.6
Latent Tuberculosis	106 (1.5)	0.6	0.5, 0.7	35 (2.5)	1.6	1.1, 2.2	2 (0.2)	0.1	0.0, 0.4
* Inflammatory bowel disease*^i^	26 (0.4)	0.1	0.1, 0.2	3 (0.2)	0.1	0.0, 0.4	17 (1.8)	0.8	0.5, 1.3
Crohn’s disease	10 (0.1)	0.1	0.0, 0.1	2 (0.1)	0.1	0.0, 0.4	7 (0.8)	0.3	0.2, 0.7
Ulcerative colitis	16 (0.2)	0.1	0.1, 0.1	1 (0.1)	0.0	0.0, 0.3	10 (1.1)	0.5	0.3, 0.9
Injection site reactions^P^	1056 (15.3)	5.9	5.5, 6.2	260 (18.6)	11.6	10.2, 13.1	156 (16.7)	7.4	6.4, 8.7
Allergic reactions/hypersensitivities	1001 (14.5)	5.6	5.2, 5.9	102 (7.3)	4.5	3.7, 5.5	88 (9.4)	4.2	3.4, 5.2
* Malignancies*	141 (2.0)	0.8	0.7, 0.9	15 (1.1)	0.7	0.4, 1.1	9 (1.0)	0.4	0.2, 0.8
NMSC	55 (0.8)	0.3	0.2, 0.4	9 (0.6)	0.4	0.2, 0.8	0 (0.0)	0.0	0.0, 0.4
Malignancies excluding NMSC	88 (1.3)	0.5	0.4, 0 .6	7 (0.5)	0.3	0.1, 0.7	9 (1.0)	0.4	0.2, 0.8
Asthma	49 (0.7)	0.3	0.2, 0.4	10 (0.7)	0.4	0.2, 0.8	5 (0.5)	0.2	0.1, 0.6
Depression and suicide/self-injury^j^	215 (3.1)	1.2	1.0, 1.4	37 (2.6)	1.6	1.2, 2.3	19 (2.0)	0.9	0.6, 1.4
MACE^k^	91 (1.3)	0.5	0.4, 0.6	12 (0.9)	0.5	0.3, 0.9	6 (0.6)	0.3	0.1, 0.6
Cytopenia^l^	171 (2.5)	0.9	0.8, 1.1	56 (4.0)	2.5	1.9, 3.2	28 (3.0)	1.3	0.9, 1.9
Iridocyclitis^m^	2 (0.0)	0.0	0.0,0.0	0 (0)	0	0.0, 0.0	58 (6.2)	2.8	2.1, 3.6

*Abbreviations: axSpA* Axial spondyloarthritis, *CI* Confidence interval, *IR* Incidence rate, *IXE* Ixekizumab, *MACE* Major adverse cerebro-cardiovascular event, *MedDRA* Medical Dictionary for Regulatory Activities, *N* Number of patients in the analysis population; n, number of patients in each category, *NMSC* Non-melanoma skin cancer, *PsA* Psoriatic arthritis, *PsO* Psoriasis, *SAE* Serious adverse event, *SMQ* Standardized MedDRA Queries *TEAE* treatment-emergent adverse event

^a^Patients with multiple occurrences of the same event are counted under the highest severity

^b^1 missing case of severity in the PsO cohort

^c^The data include 3 cases considered severe, 65 cases considered moderate, and 76 cases considered mild

^d^Data included 3 cases considered moderate and 13 cases considered mild

^e^Data included 2 cases considered moderate and 3 cases considered mild

^f^As reported by investigator

^g^Oral *Candida* infection includes oral candidiasis, oral fungal infection and oropharyngeal candidiasis

^h^PsO Cohort: Denominator adjusted because gender-specific event for females; *N* = 2196, PY = 5580.5. PsA Cohort: Denominator adjusted due to gender-specific event for females; *N* = 722, PY = 1142.2 (pooled IXE). axSpA Cohort: Denominator adjusted because gender-specific event for females; *N* = 282, PY = 592.8 (pooled IXE)

^i^Data represent adjudicated cases. For the PsO cohort, the data represents cases classified as “definite” and “probable” per external adjudication. IR was calculated as the total of “definite” and “probable” cases /total patient-years, then multiplied by 100. There were 5 cases of adjudicated IBD that were not considered TEAEs. Total adjudicated IBD *n* = 31 (0.4%, IR of 0.2 per 100 PY). 5 additional cases confirmed by adjudication occurred either on the safety follow-up period (*n* = 3) or on the placebo maintenance period after IXE treatment (*n* = 2). 3 patients with PsO had a history of IBD. For the axSpA cohort, 12 cases de novo, 5 patients had a history of IBD and experienced a flare during the study period. 1 additional case of IBD was reported in the safety follow-up

^j^Broad, according to Standardized MedDRA Queries (SMQ) or sub-SMQ classification

^k^adjudicated cases

^l^SMQ classification

^m^4 cases of uveitis were reported among patients in the PsO cohort (0.1%, IR 0.0 per 100 PY), 8 cases of uveitis were reported among patients in the axSpA cohort (0.9%, IR 0.4 per 100 PY). All studies were conducted using the original, citrate-containing formulation of IXE. AE terms were derived from MedDRA version 24.1 for PsO and axSpA-related studies, version 23.1 for PsA-related studies

^n^Most common TEAEs are defined as those with an IR > 2.0

^o^Data collection for the clinical trial database does not specify when events became serious and therefore the numbers shown may represent more serious events than what actually occurred during the treatment period

^P^Preferred MedDRA term. All studies included in these analyses used the original, citrate-containing IXE formulation. axSpA cohort includes patients with AS and nr-axSpA

#### SAEs

SAEs were reported by 969 patients with PsO (14.1%, IR 5.4 per 100 PY), 134 patients with PsA (9.6%, IR 6.0 per 100 PY), and 101 patients with axSpA (10.8%, IR 4.8 per 100 PY) who received IXE treatment (Table 2). Categories of SAEs with an IR ≥ 0.5 are listed in Table S2. Of the 969 SAEs reported among patients with PsO, 36 resulted in death (0.5%, IR 0.2 per 100 PY). Of the 134 SAEs reported among patients with PsA, 6 resulted in death (0.4%, IR 0.3 per 100 PY). Of the 101 SAEs reported among patients with axSpA, 3 resulted in death (0.3%, IR 0.1 per 100 PY). IRs of SAEs were stable over the time periods examined for patients with PsO (year 0–1: IR 6.5 per 100 PY; year 4–5: IR 5.7 per 100 PY), PsA (year 0–1: IR 6.0 per 100 PY; year 2–3: IR 5.5 per 100 PY), and axSpA (year 0–1: IR 6.2 per 100 PY; year >  = 2: IR 4.7 per 100 PY) (Fig. 1).

During the study period, 45 deaths were reported among all IXE-treated patients (36 patients with PsO, 6 patients with PsA, and 3 patients with axSpA). The causes of death in the PsO cohort were cardiovascular (*n* = 20), neoplasm (*n* = 5), unknown cause (*n* = 4), respiratory (*n* = 1), and other causes of death (*n* = 6, including 1 homicide, 1 accidental death, 1 IBD, 1 end-stage senile dementia, 1 cholelithiasis, 1 trauma). The 6 deaths reported among patients with PsA were due to cardiovascular-related events (*n* = 2), cerebrovascular accident (*n* = 1), metastatic renal cell carcinoma (*n* = 1), drowning (*n* = 1), and pneumonia (*n* = 1). The causes of death among patients with axSpA were suicide (*n* = 1), sepsis (*n* = 1), and general disorder (*n* = 1). The lengths of time of treatment among fatalities are outlined in Table S3.

#### Discontinuations from study

AEs (including death) leading to discontinuation from the study were reported among 519 patients with PsO (7.5%, IR 2.9 per 100 PY), 115 patients with PsA (8.2%, IR 5.1 per 100 PY), and 66 patients with axSpA (7.1%, IR 3.1 per 100 PY) (Table 2). The main causes of discontinuation among patients with PsO were mycobacterium TB complex positive test (*n* = 31, IR 0.2 per 100 PY), latent TB (*n* = 21, IR 0.1 per 100 PY), and maternal exposure during pregnancy (*n* = 18, IR 0.3 per 100 PY). The main causes of discontinuation among patients with PsA were a positive result on an interferon gamma release assay (*n* = 10, IR 0.4 per 100 PY) (annual test for latent TB), latent TB (*n* = 6, IR 0.3 per 100 PY) and injection site reaction (*n* = 5, IR 0.2 per 100 PY). Among patients with axSpA, infections (*n* = 13, IR 0.6 per 100 PY), injection-site reactions (*n* = 8, IR 0.4 per 100 PY), malignancies (*n* = 8, IR 0.4 per 100 PY), and IBD (confirmed by adjudication) (*n* = 7, IR 0.3 per 100 PY) were the main causes of discontinuation.

### Summary of selected adverse events

#### Infections

Infections were the most commonly reported TEAEs across indications and were mostly mild or moderate in severity (Table 2). Infections were reported among 4307 patients with PsO (62.5%, IR 23.9 per 100 PY), 759 patients with PsA (54.2%, IR 33.8 per 100 PY), and 540 patients with axSpA (57.9%, IR 25.7 per 100 PY). The most commonly reported infections (defined as IR ≥ 5.0 per 100 PY) across indications were nasopharyngitis and upper respiratory tract infection. The IRs of infections were higher in the first year of IXE exposure and did not increase over time (PsO, year 0–1 infection IR = 57.6, year 4–5 IR = 35.6; PsA, year 0–1 infection IR = 52.2, year 2–3 IR = 39.1; axSpA, year 0–1 infection IR = 49.3, year ≥ 2 IR = 28.6) (Fig. 1). The frequencies of serious infections ranged from 1.1 to 1.3 per 100 PY across indications (3.4% PsO, IR 1.3 per 100 PY; 2.0% PsA, IR 1.2 per 100 PY; 2.5% axSpA, IR 1.1 per 100 PY). Serious infections were reported among 231 patients with PsO (total of 328 events), 28 patients with PsA (total of 33 events), and 23 patients with axSpA (total of 27 events). The most common serious infections reported (IR ≥ 0.1) in the PsO cohort were cellulitis (*n* = 40, 0.6%, IR 0.2 per 100 PY), pneumonia (*n* = 28, 0.4%, IR 0.2 per 100 PY) and appendicitis (*n* = 12, 0.2%, IR 0.1 per 100 PY). In the PsA cohort, pneumonia (*n* = 6, 0.4%, IR 0.3 per 100 PY), bronchitis (*n* = 3, 0.2%, IR 0.1 per 100 PY), arthritis bacterial (*n* = 2, 0.1%, IR 0.1 per 100 PY), latent TB (*n* = 2, 0.1%, IR 0.1 per 100 PY), lower respiratory tract infection (*n* = 2, 0.1%, IR 0.1 per 100 PY) and esophageal candidiasis (*n* = 2, 0.1%, IR 0.1 per 100 PY) were the most common serious infections. In the axSpA cohort, appendicitis was the most common serious infection (*n* = 3, 0.3%, IR 0.1 per 100 PY), with 2 cases of each of the following serious infection types reported (representing 0.2% of the cohort, IR 0.1 per 100 PY for each): cellulitis, *Clostridium difficile* colitis, gastroenteritis, sinusitis, urinary tract infection and orchitis. The majority of these cases were resolved across indications (PsO *n* = 291, 88.7%; PsA *n* = 27, 81.8%; axSpA *n* = 23, 85.2%) (Table S4).

Opportunistic infections (OIs) are reported according to MedDRA PTs and occurred among 536 patients with PsO (7.8%, IR 3.0 per 100 PY), 86 patients with PsA (6.1%, IR 3.8 per 100 PY), and 28 patients with axSpA (3.0%, IR 1.3 per 100 PY). Oral candidiasis and herpes zoster were the most commonly reported OIs across indications. Oral candidiasis was reported among 144 patients with PsO (2.1%, IR 0.8 per 100 PY), 16 patients with PsA (1.1%, IR 0.7 per 100 PY), and 5 patients with axSpA (0.5%, IR 0.2 per 100 PY) (detailed description of *Candida* infections is reported below). Herpes zoster was reported among 120 patients with PsO (1.7%, IR 0.6 per 100 PY), 16 patients with PsA (1.1%, IR 0.7 per 100 PY), and 12 patients with axSpA (1.3%, IR 0.6 per 100 PY).

Latent TB infection (identified by latent TB or a positive result on an interferon-gamma release assay, mycobacterium TB complex test, or tuberculin skin test) was reported among 106 patients with PsO (1.5%, IR 0.6 per 100 PY); 47 patients had latent TB (0.7%, IR 0.3 per 100 PY), 42 patients reported a positive mycobacterium TB complex test (0.6%, IR 0.2 per 100 PY), and 17 patients demonstrated a positive tuberculin skin test (0.2%, IR 0.1 per 100 PY). In the PsA cohort, latent TB was reported in 35 patients (2.5%, IR 1.6 per 100 PY); 15 patients had latent TB (1.1%, IR 0.7 per 100 PY), 13 patients reported a positive interferon-gamma release assay (0.9%, IR 0.6 per 100 PY), 5 patients demonstrated a positive tuberculin skin test (0.4%, IR 0.2 per 100 PY), and 2 patients had a positive mycobacterium TB complex test (0.1%, IR 0.1 per 100 PY). Latent TB infection was reported among 2 patients with axSpA (0.2%, IR 0.1 per 100 PY). While latent TB infections were identified at baseline, there were no cases of active TB or reactivated TB reported.

#### Candida infections

*Candida* infections were reported in 337 patients with PsO (4.9%, IR 1.9 per 100 PY), 45 patients with PsA (3.2%, IR 2.0 per 100 PY) and 26 patients with axSpA (2.8%, IR 1.2 per 100 PY). The IR for *Candida* infections across indications was 1.8 per 100 PY*.* All cases were localized across indications, with no systemic cases reported among patients receiving IXE treatment. At least 1 event of *Candida* infection was reported in 337 patients with PsO. These cases were mainly mild (*n* = 162, IR 0.9 per 100 PY) or moderate (*n* = 170, IR 0.9 per 100 PY) in severity. Cases of severe *Candida* infection (based on investigator judgment) were reported among 5 patients with PsO (0.1%, IR 0.0 per 100 PY). At least 1 event of *Candida* infection was reported in 45 patients with PsA. These cases were mainly mild (*n* = 34, IR 1.5 per 100 PY) or moderate (*n* = 10, IR 0.4 per 100 PY) in severity. One case of severe *Candida* infection was reported in a patient with PsA (0.1%, IR 0.0 per 100 PY). At least 1 event of *Candida* infection was reported in 26 patients with axSpA. These cases were mainly mild (*n* = 16, IR 0.8 per 100 PY) or moderate (*n* = 10, IR 0.5 per 100 PY) in severity. No cases of severe *Candida* infection were reported in patients with axSpA. Oral, vulvovaginal, and skin *Candida* were the most frequently reported *Candida* infections across indications. Oral *Candida* was reported among 160 patients with PsO (2.3%, IR 0.9 per 100 PY), 22 patients with PsA (1.6%, IR 1.0 per 100 PY), and 8 patients with axSpA (0.9%, IR 0.4 per 100 PY). Vulvovaginal *Candida* was reported among 97 patients with PsO (4.4%, IR 1.7 per 100 PY) and 13 patients with PsA (1.8%, IR 1.1 per 100 PY). Vulvovaginal candidiasis was reported 7 patients with axSpA (2.5%, IR 1.2 per 100 PY). Skin *Candida* was reported among 52 patients with PsO (0.8%, IR 0.3 per 100 PY), 5 patients with PsA (0.4%, IR 0.2 per 100 PY), and 2 patients with axSpA (0.2%, IR 0.1 per 100 PY). Oesophageal candidiasis led to the discontinuation of IXE treatment for 1 patient with PsO (0.0%, IR 0.0 per 100 PY) and 1 patient with axSpA (0.1%, IR 0.0 per 100 PY). There were no discontinuations due to *Candida* infection among patients with PsA.

#### Inflammatory bowel disease

IBD cases that were classified as “definite” and “probable” per external adjudication were confirmed in 31 patients with PsO (0.5%, IR 0.2 per 100 PY) (3 patients with PsO had a history of IBD, 28 adjudicated IBD cases were de novo). This included 13 patients with Crohn’s disease (CD) (0.2%, IR 0.7 per 100 PY) and 18 patients with ulcerative colitis (UC) (0.3%, IR 0.1 per 100 PY). Five IBD cases were confirmed by adjudication either during the safety follow-up period (*n* = 3) or the placebo maintenance period, after IXE treatment (*n* = 2). One patient with ulcerative colitis had a fatal event. In the PsA cohort, 14 patients presented with a self-reported history of IBD. During the trial period, 3 cases of IBD were classified as “definite” and “probable” per external adjudication among patients with PsA receiving IXE at the time of the event (0.2%, IR 0.1 per 100 PY), including 2 cases of CD (0.1%, IR 0.1 per 100 PY) and 1 case of UC (0.1%, IR 0.0 per 100 PY). Of the 17 cases of IBD classified as “definite” and “probable” per external adjudication among patients with axSpA receiving IXE at the time of the event (1.8%, IR 0.8 per 100 PY), 7 cases were CD (0.8%, IR 0.3 per 100 PY) and 10 cases were UC (1.1%, IR 0.5 per 100 PY). 12 cases in the axSpA cohort were de novo, whereas 5 patients with axSpA had a history of IBD and experienced a flare during the study period. One additional new case of IBD was reported in this cohort during the safety follow-up period.

#### Major adverse cerebro-cardiovascular events

The (adjudicated) incidence of MACE was less than 1 per 100 PY among patients with PsO (IR 0.5 per 100 PY), PsA (IR 0.5 per 100 PY), and axSpA (IR 0.3 per 100 PY) over the treatment periods examined (Table 2). Of the 103 adjudicated MACE cases in the PsO cohort, 20 were fatal (19.4%), 57 recovered (55.3%), and 17 recovered with sequelae (16.5%). Of the 12 adjudicated MACE cases in the PsA cohort, 2 were fatal (16.7%), 9 recovered (75.0%), and 1 recovered with sequelae (8.3%). All 6 MACE cases adjudicated in the axSpA cohort recovered (100.0%). The IRs in the PsO, PsA and axSpA cohort for non-fatal myocardial infarction were 0.3 per 100 PY across all 3 indications, for non-fatal stroke were 0.1 per 100 PY (PsO) and 0.2 per 100 PY (PsA), and for cardiovascular-related death were 0.1 per 100 PY for patients in both the PsO and PsA cohorts. IRs were stable over the treatment periods (Fig. 1).

#### Malignancies

Malignancy IRs at 1-year intervals to year 5 (PsO), and up to 3 years (PsA, axSpA), remained low (≤ 1.2 per 100 PY) and constant. Malignancies occurred in 141 patients with PsO (2.0%, IR 0.8 per 100 PY), 15 patients with PsA (1.1%, IR 0.7 per 100 PY), and 9 patients with axSpA (1.0%, IR 0.4 per 100 PY) (Table 2). The mean (SD) times from the start of IXE treatment to the onset of treatment-emergent malignancy events, overall, were 670.4 (558.6) days for patients with PsO, 509.8 (284.5) days for patients with PsA, and 507.6 (383.3) days for patients with axSpA. The most commonly reported malignancy other than NMSC among the PsO and PsA cohorts was prostate cancer (IR = 0.1), and among the axSpA cohort was ovarian cancer (IR = 0.2). Non-melanoma skin cancer (NMSC) was reported among 55 patients with PsO (IR 0.3 per 100 PY), 9 patients with PsA (IR 0.4 per 100 PY), and no cases reported in patients with axSpA. Among patients with NMSC, IRs of basal cell carcinoma and squamous cell carcinoma were the highest reported, across indications (Table S5).

#### Injection site reactions

After infections, ISRs were the second most common selected AE of interest across indications. ISRs were reported among 1,056 patients with PsO (15.3%, IR 5.9 per 100 PY), 260 patients with PsA (18.6%, IR 11.6 per 100 PY), and 156 patients with axSpA (16.7%, IR 7.4 per 100 PY) (Table 2). The majority of ISRs reported in the PsO cohort were mild (*n* = 727 [10.5%], IR 4.0 per 100 PY) or moderate (*n* = 294 [4.3%], IR 1.6 per 100 PY). Thirty-five patients with PsO (0.5%, IR 0.2 per 100 PY) experienced a severe ISR. The majority of ISRs reported in the PsA cohort were mild (*n* = 207 [14.8%], IR 9.2 per 100 PY) or moderate (*n* = 48 [3.4%], IR 2.1 per 100 PY). Five patients with PsA (0.4%, IR 0.2 per 100 PY) experienced a severe ISR. In the axSpA cohort, 115 patients experienced mild ISRs (12.3%, IR 5.5 per 100 PY, 35 patients experienced moderate ISRs (3.8%, IR 1.7 per 100 PY), and 6 patients experienced severe ISRs (0.6%, IR 0.3 per 100 PY). IRs of ISRs decreased over the time periods examined across indications (PsO, IR 16.5 (year 0–1) to 1.7 (year 4–5); PsA, IR 21.3 (year 0–1) to 2.3 (year 2–3); axSpA, IR 18.2 (year 0–1) to 1.9 (year ≥ 2). ISRs led to the discontinuation of IXE treatment for 15 patients with PsO (0.2%, IR 0.1 per 100 PY), 9 patients with PsA (0.6%, IR 0.4 per 100 PY), and 8 patients with axSpA (0.9%, IR 0.4 per 100 PY). All studies included in these analyses used the original, citrate-containing IXE formulation and not the new citrate-free formulation, which demonstrates less frequent reports of ISRs and less injection site pain, post-injection [28].

#### Depression and suicide/self-injury

Depression and suicide/self-injury (broad term used according to Standardized MedDRA Queries (SMQ) or sub-SMQ classification) were reported among 215 patients with PsO (3.1%, IR 1.2 per 100 PY), 37 patients with PsA (2.6%, IR 1.6 per 100 PY), and 19 patients with axSpA (2.0%, IR 0.9 per 100 PY) (Table 2). The most common subcategories among the entire PsO cohort were depression (*n* = 132, 1.9%, IR 0.7 per 100 PY), depressed mood (*n* = 11, 0.2%, IR 0.1 per 100 PY), and suicide attempt (*n* = 11, 0.2%, IR 0.1 per 100 PY). Among patients with PsA, these subcategories were depression (*n* = 24, 1.7%, IR 1.1 per 100 PY), depressed mood (*n* = 6, 0.4%, IR 0.3 per 100 PY), and adjustment disorder with depressed mood (*n* = 2, 0.1%, IR 0.1 per 100 PY). Depression (*n* = 11, 1.2%, IR 0.5 per 100 PY), suicidal ideation (*n* = 2, 0.2%, IR 0.1 per 100 PY), and completed suicide (*n* = 1, 0.1%, IR 0.0 per 100 PY) were the most common subcategories reported among patients with axSpA. The IRs of depression decreased over time, across all indications (year 0–1, PsO IR 1.7 to 0.7, year 4–5; year 0–1 PsA, IR 2.2 to 0.3, year 2–3; year 0–1, axSpA IR 1.1 to 0.7 year >  = 2].

#### Other selected AEs of interest

Iridocyclitis was reported in 2 patients with PsO (0.0%, IR 0.0 per 100 PY), while no cases were reported among patients with PsA. Iridocyclitis was reported in 58 patients with axSpA (6.2%, IR 2.8 per 100 PY) (Table 2), 43 patients in the overall axSpA cohort (4.6%, IR 2.1 per 100 PY) had a history of iridocyclitis. Uveitis was reported in 4 patients with PsO (0.1%, IR 0.0 per 100 PY) and 8 patients with axSpA (0.9%, IR 0.4 per 100 PY). No cases of uveitis were reported among patients with PsA. Selected AEs of interest were additionally examined according to sex and can be found in the supplement (Table S6).

## Discussion

Due to the chronic nature of PsO, PsA, and axSpA, long-term treatment and concurrent safety monitoring are required. In this end-of-study program update to previously disclosed data [12], we report safety results pooled from 9,225 patients (embodying data from an additional 997 patients from the previous report [12]) who received IXE treatment for PsO, PsA or axSpA (including AS and nr-axSpA) in 25 RCTs. Overall, the median IXE exposure duration ranged between 1.3 and 2.7 years across the PsO, PsA, and axSpA populations, reflective of patterns of utilization observed in real-world clinical practice [29, 30]. The IRs of TEAEs, SAEs, and selected AEs of interest are reported.

Long-term exposure to IXE did not increase the IRs of TEAEs or SAEs. Infections and ISRs were the most common TEAEs observed and were most frequently reported during the first year of IXE treatment. The IRs of infections and ISRs did not increase over time, and the majority were mild or moderate in severity across all indications, results consistent with previous IXE-related safety reports [12, 16, 18, 31]. The studies included in the current analyses used the original IXE formulation and not the new citrate-free formulation, which was developed to improve overall patient experiences (demonstrating less frequent reports of ISRs and less injection site pain, post-injection [28]). The IRs of SAEs were similar across indications and did not increase over time. The frequencies of AEs leading to discontinuation were similar across indications.

Concerns surrounding the safety of immunomodulators due to an associated increased risk of infections have been raised previously [32–35]. Our findings reveal that although infections were the most common TEAEs across indications and the most frequent reason for discontinuation, the severity of infections were mostly mild or moderate. Upper respiratory tract infections and other common infections (sinusitis, etc.; Table 2) were the most frequently reported PTs. The frequencies of serious infections were low across indications. Evaluation of safety data (particularly data collected from non-controlled portions of clinical trials) is aided by consideration of findings from other clinical development programs and the background incidence in the target populations of interest. Although direct comparisons among data sources are complicated by differences in patient populations, data collection, and time periods, examination of external data sources provides context for what may reasonably be expected in the intended patient population. The incidences of serious infections observed in this analysis (PsO IR 1.3, PsA IR 1.2, axSpA IR 1.1 per 100 PY) are similar to those reported in PsO, PsA, and axSpA clinical development programs of another IL-17 inhibitor [36] (PsO IR 1.4, PsA IR 1.8, axSpA IR 1.2 per 100 PY) and a TNF inhibitor [37] (PsO IR 1.8, PsA IR 2.8, axSpA IR 1.0 per 100 PY) and these are also similar to reports from real-world PsO [38], PsA [39], and axSpA populations [40].

The majority of cases of serious infections resolved (PsO *n* = 291, 88.7%; PsA *n* = 27, 81.8%; axSpA *n* = 23, 85.2%). Severe infections were infrequent. OIs were largely due to *Candida* species, likely due to the role of IL-17 in host defense against infections of this type [41]. There is evidence to suggest that patients with PsO have an increased risk of *Candida* colonization (odds ratio: 2.47; 95% CI: 1.68 – 3.6 [42]) compared to control individuals, and the rate of oral candidiasis is higher among patients with PsO compared to the general population [43]. No relevant information on the background rate of *Candida* infection is available in the general PsA or axSpA populations. Reported cases of *Candida* infection in the current study were mainly mild or moderate in severity across indications. Severe cases of *Candida* infection were infrequent. Two cases of esophageal candidiasis led to the discontinuation of IXE treatment for 1 patient with PsO and 1 patient with axSpA. IRs of *Candida* infection in the current study were similar to those reported following use of another IL-17A inhibitor in patients with PsO, PsA and axSpA [36], and although different members of the IL-17 inhibitor family demonstrate different rates of *Candida* infection [36, 44, 45], we demonstrate consistently low rates over long-term use with IXE. Among patients with reported candida infection treatments, topical anti-fungal treatment was the most common.

Relative to the general population, patients with PsO, PsA, and axSpA have an increased (1–fourfold) risk of developing IBD [46–48]. IBD events reported in the current study were uncommon (< 2% of the patient population), with 26 cases reported among patients with PsO (0.4%, IR 0.1 per 100 PY), 3 cases reported among patients with PsA (0.2%, IR 0.1 per 100 PY) and 17 cases reported among patients with axSpA (1.8%, IR 0.8 per 100 PY). In the present analysis, the IR of reported IBD (including UC and CD) overall ranged from 0.1 to 0.8 per 100 PY across the disease states examined, which is within the range of background rates observed for each disease in the general population (UC: 0.02-0.31 [PsO]; 0.03-0.11 [PsA]; 1.05 [axSpA]) (CD: 0.02-0.25 [PsO]; 0.04-0.06 [PsA]; 0.99 [axSpA]) [46, 49–52]. Smoking, obesity, infections, and high doses of NSAIDs have previously been noted as risk factors associated with bowel inflammation [53–57]. A sizable portion of the axSpA (29.6%) and PsA (38.4%) cohorts were smokers at the time of the trials, 12.7% of the PsO cohort were smokers. A meta-analysis previously revealed that smokers have an increased risk of developing Crohn disease and a reduced risk of developing ulcerative colitis (compared with former smokers) [58]. In this study, a majority of patients were non-smokers, therefore it is difficult to conclude whether smoking status influenced the rates reported for ulcerative colitis. The mean BMI of patients in the PsO (30.4 kg/m2) and PsA cohorts (30.0 kg/m2) fell within the range considered as adult obesity, the mean value for patients in axSpA cohort (27.5 kg/m2) was representative of overweight adults [49, 59–62].

In the current study, the incidence of MACE events were low across patient cohorts (≤ 0.5 per 100 PY). These findings are similar to those reported in clinical development programs of other biologic medications within similar target populations (IR MACE PsO = 0.4, PsA = 0.4, AS = 0.7 per 100 PY [36]; IR MACE PsO = 0.3 per 100 PY [63]) and those observed in real-world patient registries of psoriasis patients (IR MACE PsO = 0.6–0.8 per 100 PY [64], IR MACE 0.2–0.4 depending on drug exposure group [65]. Registry data is limited in the PsA and axSpA populations; however, the incidence of MACE observed in the clinical development program of those target populations is within the range of rates reported in published observational literature [3, 66, 67]. The low and stable incidence of MACE is particularly encouraging for the long-term treatment of patients across the indications examined, as cardiovascular risk factors are among the most common comorbidities associated with PsO, owing in part to the prolonged duration of exposure to inflammatory factors associated with vascular diseases [68, 69]. Similarly, PsA and axSpA also confer an increased risk of cardiovascular disease; therefore, the low, and stable incidence of MACE observed throughout the IXE treatment period in this program, highlights IXE as a viable therapeutic treatment option for patients with PsO, PsA, and axSpA.

In the current study, malignancies were reported by 2.0% of patients with PsO, with an IR of 0.8 per 100 PY and by 1.1% of patients with PsA, with an IR of 0.7 per 100 PY. The IR of malignancy observed among axSpA patients exposed to IXE was 0.4 per 100 PY. These rates are similar to those reported in clinical development programs of other biologics [36, 37] and in real-world registries with psoriasis and other rheumatic diseases [70, 71], including a recent meta-analysis that reported the IR of cancer in patients with PsO as 1.75 per 100 PY and in patients with PsA, 0.64 per 100 PY [72]. As part of a follow-up study to this investigation, the incidence of malignancy (excluding NMSC) observed in the IXE clinical development program, for all three indications, was compared to the incidence of malignancy in the general population, using the SEER Cancer Registry [2013–2017], standardized by age and sex. Standardized incidence ratios generated showed that the occurrence of malignancy in the clinical development program was similar to what would be expected in the general population given the age and sex-specific rates in the general US population. In addition, this post-hoc analysis revealed that among patients who had a treatment-emergent malignancy event, the mean time to onset of malignancy events from the start of IXE treatment was 1.8 years for patients with PsO, and 1.4 years for patients with PsA and axSpA. Given the long latency of solid cancers associated with exposure to known carcinogens [73], it is unlikely that the treatment-emergent malignancy events observed in this investigation are due to IXE treatment.

Although this study covers up to 2,247.7 PY of exposure to IXE for patients with PsA, and 2,097.7 PY of exposure for patients with axSpA, the duration of the study periods and the relatively small patient sample size in these cohorts are limitations to this study. We also acknowledge the disparity among the numbers of patients representing different races, with a predominantly white patient population represented across indications (> 70% of participants) and limited number of Black/African American, Hispanic, and Asian patients included. While pooling of patient data across studies has facilitated a comprehensive overview of safety outcomes across cohorts, patient characteristics, baseline selection criteria, treatment regimens, and doses differ between the studies included in this integrated data set. Additionally, an inability to stratify risk exists, based on the severity/control of comorbid conditions. The interpretation of TB test results is difficult without a detailed insight into the prevalence of TB in the relevant populations. The lack of a long-term comparator in this study limits any direct comparisons of IRs to those observed following the use of different therapeutic options, however these factors resemble real-world treatment plans. Furthermore, like any study of its kind, we must acknowledge the potential for survivor bias over the course of long-term IXE treatment, where individuals most prone to AEs early in the treatment regimen may discontinue, leaving individuals who were less likely to have an AE in the remaining patient pool. Even so, the lack of an increasing trend in the IRs observed during follow-up across PsO, PsA and axSpA populations supports that there is no association with events that require prolonged exposure or have a longer latency.

## Conclusion

The large patient population assessed in this study, pooled from 25 RCTs, across multiple indications provides a comprehensive update to previously published data relating to the safety of long-term use of IXE in patients with PsO, PsA, and axSpA [12]. The robustness of the results presented is enhanced by use of IRs adjusted for exposure. No new or unexpected safety signals were found in this study, with rates of safety events of special interest (including serious infections, IBD, ISR, MACE, malignancy) remaining stable through longer exposure with IXE treatment and within the expected ranges [12, 16, 18, 31, 36, 37, 63, 69–71, 74] in patients across the indications examined. These final, end-of-study program results surrounding the long-term use of IXE in patients with PsO, PsA and axSpA should serve as an important point of reference for physicians considering IXE treatment in these patients, over an extended period.

### Supplementary Information

**Additional file 1: Supplementary material. Table S1:** Overview of clinical trials in this study and patient baseline risk relevant to safety. **Figure S1:** Cumulative and maximum IXE exposure. **Table S2:** Categories of serious adverse events. **Table S3:** Duration of IXE exposure (days) among fatalities. **Table S4:** Outcomes for infections and serious infections in patients across indications. **Table S5:** IRs per 100 PY for malignancies across indications, over the time periods examined. **Table S6:** Selected AEs of interest examined by sex.

**Additional file 2. **Study Infographic. Overview of Safety Outcomes across Indications.

## Data Availability

The datasets generated and/or analyzed during this study are available on reasonable request.

## Abbreviations

AE: Adverse event
AS: Ankylosing spondylitis
ASAS: Assessment of Spondyloarthritis International Society
axSpA: Axial spondyloarthritis
BASDAI: Bath Ankylosing Spondylitis Disease Activity Index
BCC: Basal cell carcinoma
bDMARD: Biologic disease-modifying anti-rheumatic drug
BMI: Body mass index
BSA: Body surface area
CASPAR: Classification for Psoriatic Arthritis
CD: Crohn’s disease
CI: Confidence interval
COPD: Chronic obstructive pulmonary disease
COVID-19: Coronavirus disease 2019
CRP: C-reactive protein
CS: Corticosteroid
csDMARD: Conventional synthetic disease-modifying anti-rheumatic drug
DLQI: Dermatology Life Quality Index
EAIR: Exposure-adjusted incidence rate
EP: Erythrodermic psoriasis
GPP: Generalized pustular psoriasis
IBD: Inflammatory bowel disease
IL: Interleukin
IR: Incidence rate
ISR: Injection site reaction
IXE: Ixekizumab
MACE: Major adverse cerebro-cardiovascular events
MedDRA: Medical Dictionary for Regulatory Activities
MHLW: Ministry of Health
MRI: Magnetic resonance imaging
NMSC: Non-melanoma skin cancer
nr-axSpA: Non-radiographic axial spondyloarthritis
NRS: Numeric rating scale
NSAID: Non-steroidal anti-inflammatory drugs
OI: Opportunistic infection
PASI: Psoriasis Area and Severity Index
PBO: Placebo
Ps: Plaque psoriasis
PsA: Psoriatic arthritis
PsO: Psoriasis
PT: Preferred term
PY: Patient-years
RCT: Randomized clinical trials
SAE: Serious adverse event
SCC: Squamous cell carcinoma
SD: Standard deviation
SMQ: Standardized MedDRA Queries
sPGA: Static Physician Global Assessment
TB: Tuberculosis
TEAE: Treatment-emergent adverse event
TNF: Tumor necrosis factor
UC: Ulcerative colitis
