# Supplementary material for: Long-term safety of Ixekizumab in adults with psoriasis, psoriatic arthritis, or axial spondyloarthritis: a post-hoc analysis of final safety data from 25 randomized clinical trials
Source: Arthritis Res Ther. 2024 Feb 12;26:49. doi: 10.1186/s13075-023-03257-7 (PMC10860236; doi:10.1186/s13075-023-03257-7)
Supplement: Supplementary file 1 — Additional file 1: Supplementary material. Table S1: Overview of clinical trials in this study and patient baseline risk relevant to safety. Figure S1: Cumulative and maximum IXE exposure. Table S2: Categories of serious adverse events. Table S3: Duration of IXE exposure (days) among fatalities. Table S4: Outcomes for infections and serious infections in patients across indications. Table S5: IRs per 100 PY for malignancies across indications, over the time periods examined. Table S6: Selected AEs of interest examined by sex. [file 13075_2023_3257_MOESM1_ESM.docx]

**Long-term Safety of Ixekizumab in Adults with Psoriasis, Psoriatic Arthritis, or Axial Spondyloarthritis: A Post-hoc Analysis of Final Safety Data from 25 Randomized Clinical Trials**

**Supplementary Material**

**Table S1:** Overview of clinical trials in this study and patient baseline risk relevant to safety page 2

**Figure S1:** Cumulative and maximum IXE exposure page 11

**Table S2**: Categories of serious adverse events page 12

**Table S3**: Duration of IXE exposure (days) among fatalities page 13

**Table S4:** Outcomes for infections and serious infections in patients across indications page 14

**Table S5:** IRs per 100 PY for malignancies across indications, over the time periods examined page 15

**Table S6:** Selected AEs of interest examined by sex page 16

**Table S1:** Overview of clinical trials in this study and patient baseline risk relevant to safety

| **Trial Phase** | **PsO IXE**  **(N=6892)** | **PsO Cohort**  **Baseline risk** | **PsA IXE**  **(N=1401)** | **PsA Cohort**  **Baseline risk** | **axSpA IXE**  **(N=932)** | **axSpA Cohort**  **Baseline risk** |
| --- | --- | --- | --- | --- | --- | --- |
| **I** | **I1F-MC-RHAG** | (i) Men or women ≥18 and <65 years of age with chronic psoriasis vulgaris for at least 6 months prior to randomization  (ii) Have plaque psoriasis (Ps) with ≥15% body surface area (BSA)  (iii) Have a Psoriasis Area and Severity Index (PASI) total score of ≥13 | - | - | - | - |
|  | **I1F-MC-RHBN**  **(NCT03073213)** | (i) Present with chronic Ps based on a confirmed diagnosis of chronic psoriasis vulgaris for at least 6 months before baseline  (ii) Have ≥10% BSA involvement at screening (Visit 1) and baseline (Week 0; Visit 2)  (iii) Have a BMI between 18 and 35, inclusive  (iv) Have both an sPGA score of ≥3 and a PASI score of ≥12 at screening (Visit 1) and baseline (Week 0; Visit 2)  (v) Are candidates for phototherapy and/or systemic therapy at the discretion of the investigator. | - | - | - | - |
|  | **I1F-MC-RHBU**  **(NCT02993471)** | (i) Present with chronic Ps based on a confirmed diagnosis of chronic psoriasis vulgaris for at least 6 months prior to screening  (ii) Have ≥10% BSA involvement at screening and first admission  (iii) Have a BMI of between 18.5 and 40.0 kg/m^2^, inclusive, at screening  (iv) Are candidates for phototherapy and/or systemic therapy at the discretion of the investigator. | - | - | - | - |
| **II** | **I1F-MC-RHAJ**  **(NCT01107457)** | (i) Present with chronic Ps based on a diagnosis of chronic psoriasis vulgaris for at least 6 months prior to randomization  (ii) At least 10% BSA involvement and a PASI score of at least 12 at Visits 1 and 2  (iii) Are candidates for systemic therapy  (iv) Have an sPGA score of at least 3 at Visits 1 and 2. | - | - | - | - |
| **III** | **UNCOVER-1**  **(NCT01474512)** | (i) Present with chronic Ps based on a confirmed diagnosis of chronic psoriasis vulgaris for at least 6 months prior to randomization  (ii) At least 10% BSA of PsO at screening and randomization  (iii) An sPGA score of at least 3 and a PASI score of at least 12 at screening and randomization  (iv) Are candidates for phototherapy and/or systemic therapy. | **SPIRIT-P1**  **(NCT01695239)** | (i) Present with an established diagnosis of active PsA for at least 6 months and currently meet CASPAR criteria  (ii) Active PsA defined as the presence of at least 3 tender and at least 3 swollen joints  (iii) Presence of active psoriatic skin lesion or a personal history of Ps. | **COAST-V**  **(NCT02696785)** | (i) Are ambulatory patients  (ii) Diagnosis of rad‑xSpA with sacroiliitis defined radiographically according to the modified New York criteria  (iii) Have a history of back pain ≥3 months with age at onset <45 years  (iv) In the past, had an inadequate response to at least 2 NSAIDs (for a duration of 4 weeks) or cannot tolerate NSAIDs  (v) If taking NSAIDs, be on a stable dose for at least 2 weeks prior to randomization  (vi) Have a history of prior therapy for axSpA for at least 12 weeks prior to screening. |
|  | **UNCOVER-2**  **(NCT01597245)** | (i) Present with chronic Ps based on a confirmed diagnosis of chronic plaque psoriasis for at least 6 months prior to first dose of study drug  (ii) At least 10% BSA of PsO at screening and first dose of study drug  (iii) An sPGA score of at least 3 and a PASI score of at least 12 at screening and first dose of study drug  (iv) Are candidates for phototherapy and/or systemic therapy. | **SPIRIT-P2**  **(NCT02349295)** | (i) Present with an established diagnosis of active PsA for at least 6 months and currently meet CASPAR criteria  (ii) Active PsA defined as the presence of at least 3 tender and at least 3 swollen joints  (iii) Presence of active psoriatic skin lesion or a history of Ps  (iv) Have been treated with 1 or more cDMARDs  (v) Have had prior treatment with at least 1 and not more than 2 TNF inhibitors (the participant must have discontinued at least 1 TNF inhibitor due to either an inadequate response [based on a minimum of 12 weeks on therapy] or documented intolerance). | **COAST-W**  **(NCT02696798)** | (i) Are ambulatory patients  (ii) Diagnosis of rad-xSpA with sacroiliitis defined radiographically according to the modified New York criteria  (iii) Have a history of back pain ≥3 months with age at onset <45 years  (iv) In the past, had an inadequate response to at least 2 NSAIDs (for a duration of 4 weeks) or cannot tolerate NSAIDs  (v) If taking NSAIDs, be on a stable dose for at least 2 weeks prior to randomization  (vi) Have a history of prior therapy for axSpA for at least 12 weeks prior to screening. |
|  | **UNCOVER-3**  **(NCT01646177)** | (i) Present with chronic Ps based on a confirmed diagnosis of chronic PsO for at least 6 months prior to randomization  (ii) At least 10% BSA of PsO at screening and randomization  (iii) An sPGA score of at least 3 and a PASI score of at least 12 at screening and randomization  (iv) Are candidates for phototherapy and/or systemic therapy. | **SPIRIT-P3**  **(NCT02584855)** | (i) Present with an established diagnosis of active PsA for at least 6 months and currently meet CASPAR criteria  (ii) Active PsA defined as the presence of at least 3 tender and at least 3 swollen joints  (iii) Presence of active psoriatic skin lesion or a history of Ps  (iv) Have been treated with 1 or more cDMARDs. | **COAST-X**  **(NCT02757352)** | (i) Are ambulatory patients  (ii) Diagnosis of nr-axSpA and fulfillment of the 2009 ASAS classification criteria  (iii) Have a history of back pain ≥3 months with age at onset <45 years  (iv) Have active nr-axSpA defined as BASDAI ≥4 and total back pain ≥4 on an NRS at screening and baseline  (v) Have objective signs of inflammation by the presence of sacroiliitis on MRI and/or presence of elevated CRP  (vi) In the past, had an inadequate response to at least 2 NSAIDs (for a duration of 4 weeks) or cannot tolerate NSAIDs  (vii) If taking NSAIDs, be on stable dose for at least 2 weeks prior to randomization  (viii) Have a history of prior therapy for axSpA for at least 12 weeks prior to screening |
|  | **IXORA-P** | (i) Have a diagnosis of moderate-to-severe plaque‑type PsO for at least 6 months prior to baseline as determined by the investigator  (ii) Have a PASI score of ≥12, an sPGA score of ≥3, and ≥10% BSA involvement at screening and baseline  (iii) Are candidates for phototherapy or systemic treatment or considered by the investigator as not adequately controlled by topical therapies. | - | - | **COAST-Y**  **(NCT03129100)** | Have completed the final study visit in Studies RHBV (NCT02696785), RHBW (NCT02696798), or RHBX (NCT02757352)  (Note: Participants from Study RHBX are not eligible if they permanently discontinued IXE and were receiving a TNF inhibitor.) |
|  | **IXORA-S**  **(NCT02561806)** | (i) Chronic Ps for at least 6 months before baseline  (ii) Failure, contraindication, or intolerability to at least 1 systemic therapy (including cyclosporine, methotrexate, or phototherapy)  (iii) A PASI score of at least 10 at screening and baseline. | - | - | - | - |
|  | **IXORA-Q**  **(NCT02718898)** | (i) Have chronic Ps based on a diagnosis of chronic Ps for at least 6 months before baseline  (ii) Have moderate-to-severe PsO in the genital area at screening and baseline  (iii) Have PsO in a nongenital area at screening and baseline  (iv) Have failed to respond to, or are intolerant of, at least 1 topical therapy used for treatment of PsO affecting the genital area. | - | - | - | - |
|  | **UNCOVER-A** | (i) Have a confirmed diagnosis of PsO for at least 6 months prior to baseline  (ii) Are candidates for phototherapy and/or systemic therapy  (iii) Have 10% BSA involvement  (iv) A PASI score of at least 12 at screening (visit 1) and at baseline (week 0; visit 2)  (v) Have an sPGA score of at least 3 | - | - | - | - |
|  | **UNCOVER-J**  **(NCT01624233)** | (i) Present with chronic Ps based on a confirmed diagnosis of PsO for at least 6 months prior to enrollment  (ii) At least 10% BSA of PsO at screening and enrollment for participants with plaque psoriasis (Ps)  (iii) An sPGA score of at least 3 and a PASI score of at least 12 at screening and enrollment for participants with Ps  (iv) Are candidates for phototherapy and/or systemic therapy. | - | - | - | - |
|  | **I1F-MC-RHBH**  **(NCT03364309)** | (i) Present with chronic Ps based on a confirmed diagnosis of chronic psoriasis vulgaris for at least 6 months prior to baseline  (ii) Have ≥10% BSA involvement at screening and baseline  (iii) Have both an sPGA score of ≥3 and a PASI score of ≥12 at screening and baseline  (iv) Are candidates for phototherapy and/or systemic therapy. | - | - | - | - |
|  | **I1F-US-RHBO**  **(NCT02387801)** | (i) Present with chronic moderate-to-severe Ps based on a confirmed (by a dermatologist) diagnosis of chronic Ps for at least 6 months prior to baseline  (ii) Active psoriatic skin lesions of Ps  (iii) Are candidates for phototherapy and/or systemic therapy. | - | - | - | - |
|  | **I1F-EW-RHBZ**  **(NCT02634801)** | (i) Present with moderate‑to‑severe chronic Ps based on a diagnosis of chronic PsO for at least 6 months before baseline  (ii) Participants who are candidates for systemic therapy and who are naive to systemic treatment for PsO  (iii) Have a either a PASI score of >10 or BSA involvement of >10% and a DLQI score of >10 at screening and baseline. | - | - | - | - |
| **IV** | **IXORA-R**  **(NCT03573323)** | (i) Have chronic Ps based on a diagnosis for at least 6 months before baseline as determined by the investigator  (ii) Are candidates for phototherapy and/or systemic therapy  (iii) Have both an sPGA score of ≥3 and a PASI score of ≥12 at screening and baseline  (iv) Have ≥10% BSA involvement at screening and baseline. | **SPIRIT-H2H^a^**  **(NCT03151551)** | (i) Presence of an established diagnosis of active PsA for at least 6 months and currently meet CASPAR criteria  (ii) Active PsA defined as the presence of at least 3 (out of 68) tender and at least 3 (out of 66) swollen joints  (iii) Presence of active Ps with ≥3% BSA involvement  (iv) Have had an inadequate response when treated with 1 or more csDMARDs. | - | - |
|  | **I1F-JE-RHCV**  **(NCT03942042)** | (i) Present with GPP or EP based on an investigator‑confirmed diagnosis and meet the associated criteria  GPP: Meet the criteria for GPP set by the MHLW at screening and baseline regardless of IL‑36 mutation status  EP: Diagnosed to have ≥80% BSA involvement (with inflammatory erythema) at screening and baseline. | - | - | - | - |

^a^ Phase 3b/4 study

ASAS, Assessment of Spondyloarthritis International Society; axSpA, axial spondyloarthritis; BASDAI, Bath Ankylosing Spondylitis Disease Activity Index; BMI, body mass index; BSA, body surface area; CASPAR, Classification for Psoriatic Arthritis; cDMARD, conventional disease-modifying antirheumatic drug; CRP, C‑reactive protein; csDMARD, conventional synthetic disease-modifying antirheumatic drug; DLQI, Dermatology Life Quality Index; EP, erythrodermic psoriasis; GPP, generalized pustular psoriasis; IXE, ixekizumab; MHLW, Ministry of Health, Labour and Welfare; MRI, magnetic resonance imaging; nr-axSpA, N, number of patients in the analysis population; nonradiographic axial spondyloarthritis; NRS, numeric rating scale; NSAID, nonsteroidal anti‑inflammatory drug; PASI, Psoriasis Area and Severity Index; Ps, plaque psoriasis; PsA, psoriatic arthritis; PsO, psoriasis; rad‑xSpA, radiographic axial spondyloarthritis; sPGA, Static Physician Global Assessment; TNF, tumor necrosis factor.

**Figure S1:** Cumulative and maximum IXE exposure

^a^Maximum IXE exposure for individual patients across the pooled trials

axSpA, axial spondyloarthritis; IXE, ixekizumab; N, number of patients in the analysis population; PsA, psoriatic arthritis; PsO, psoriasis; PY, patient-years

**Table S2:** Categories of serious adverse events with IR ≥0.5

| **Category of SAE (IR ≥0.5)** | **Integrated PsO IXE (N=6892)** | **Integrated PsA IXE**  **(N=1401)** | **Integrated axSpA IXE**  **(N=932)** |
| --- | --- | --- | --- |
|  | IR per 100 PY | IR per 100 PY | IR per 100 PY |
| Infections and infestations | 1.3 | 1.2 | 1.1 |
| Cardiac disorders | 0.7 | 0.8 | 0.5 |
| Injury, poisoning and procedural complications | 0.7 | 0.8 | 0.6 |
| Gastrointestinal disorders | 0.6 | 0.7 | 0.6 |
| Neoplasms benign, malignant and unspecified (including cysts and polyps) | 0.6 | 0.6 | 0.6 |
| Musculoskeletal and connective tissue disorders | 0.5 | 0.5 | 0.8 |

Note: Patients with multiple occurrences of these categories are counted once for each category. Patients may

be counted in more than one category. axSpA, axial spondyloarthritis; IXE, ixekizumab; PsA, psoriatic arthritis; PsO,

psoriasis; SAE, serious adverse event.

**Table S3:** Duration of IXE exposure (days) among fatalities

| **Pooled PsO IXE Deaths**  **(N=6892)** | | **Pooled PsA IXE Deaths**  **(N=1401)** | | **Pooled axSpA IXE Deaths**  **(N=932)** | |
| --- | --- | --- | --- | --- | --- |
| **Cause of death** | **Duration of IXE exposure (days)** | **Cause of death** | **Duration of IXE exposure (days)** | **Cause of death** | **Duration of IXE exposure (days)** |
| Cardiovascular-related event | 65 | Cardiovascular-related event | 394 | Sepsis | 759 |
| Cardiovascular-related event | 128 | Cardiovascular-related event | 642 | General disorder | 289 |
| Cardiovascular-related event | 171 | Cerebrovascular accident | 537 | Suicide | 54 |
| Cardiovascular-related event | 246 | Metastatic renal cell carcinoma | 923 |  |  |
| Cardiovascular-related event | 432 | Pneumonia | 19 |  |  |
| Cardiovascular-related event | 454 | Drowning | 192 |  |  |
| Cardiovascular-related event | 577 |  |  |  |  |
| Cardiovascular-related event | 759 |  |  |  |  |
| Cardiovascular-related event | 900 |  |  |  |  |
| Cardiovascular-related event | 1154 |  |  |  |  |
| Cardiovascular-related event | 1159 |  |  |  |  |
| Cardiovascular-related event | 1174 |  |  |  |  |
| Cardiovascular-related event | 1231 |  |  |  |  |
| Cardiovascular-related event | 1602 |  |  |  |  |
| Neoplasms | 299 |  |  |  |  |
| Neoplasms | 949 |  |  |  |  |
| Neoplasms | 1085 |  |  |  |  |
| Neoplasms | 1676 |  |  |  |  |
| Neoplasms | 1746 |  |  |  |  |
| Unknown cause | 134 |  |  |  |  |
| Unknown cause | 1072 |  |  |  |  |
| Unknown cause | 1126 |  |  |  |  |
| Unknown cause | 1426 |  |  |  |  |
| Respiratory failure | 786 |  |  |  |  |
| Respiratory failure | 863 |  |  |  |  |
| Respiratory failure | 1556 |  |  |  |  |
| Nervous system disorders | 219 |  |  |  |  |
| Nervous system disorders | 620 |  |  |  |  |
| Vascular disorder | 129 |  |  |  |  |
| Hepatobiliary disorder | 1652 |  |  |  |  |
| Gastrointestinal disorder | 1480 |  |  |  |  |
| Infections and infestations | 1233 |  |  |  |  |
| General disorder | 877 |  |  |  |  |
| Accidental death | 462 |  |  |  |  |
| Victim of homicide | 1538 |  |  |  |  |
| Trauma | 68 |  |  |  |  |

Time (i.e., duration of exposure) in days at the time the event starts relative to the treatment period start date

axSpA, axial spondyloarthritis; IXE, ixekizumab; N, number of patients in the analysis population; PsA, psoriatic arthritis; PsO, psoriasis.

**Table S4:** Outcomes for infections and serious infections in patients across indications

|  | **Patient with ≥1 TEAE**  **n (%)^a^** | **Total number of events**  **Nx** | **Fatal**  **n (%)^b^** | **Recovered/resolved**  **n (%)^b^** | **Not recovered/not resolved**  **n (%)^b^** | **Recovered/resolved with sequelae**  **n (%)^b^** | **Recovering/resolving**  **n (%)^b^** | **Unknown**  **n (%)^b^** | **Missing**  **n (%)^b^** |
| --- | --- | --- | --- | --- | --- | --- | --- | --- | --- |
| **Infections**  PsO  PsA  axSpA | 4307 (62.5)  759 (54.2)  540 (57.9) | 14146  1923  1423 | 1 (0.0)  1 (0.1)  1 (0.1) | 13385 (94.6)  1858 (96.6)  1347 (94.7) | 470 (3.3)  33 (1.7)  41 (2.9) | 63 (0.4)  6 (0.3)  1 (0.1) | 180 (1.3)  19 (1.0)  33 (2.3) | 29 (0.2)  4 (0.2)  0 | 18 (0.1)  2 (0.1)  0 |
| **Serious infections**  PsO  PsA  axSpA | 231 (3.4)  28 (2.0)  23 (2.5) | 328  33  27 | 1 (0.3)  1 (3.0)  1 (3.7) | 291 (88.7)  27 (81.8)  23 (85.2) | 18 (5.5)  1 (3.0)  0 | 12 (3.7)  3 (9.1)  0 | 4 (1.2)  1 (3.0)  3 (11.1) | 2 (0.6)  0  0 | 0  0  0 |

^a^ Percentage was calculated by n/N*100%.

^b^ Percentage was calculated by n/Nx*100%.

axSpA, axial spondyloarthritis; N, number of patients in the analysis population; n, number of patients each category; Nx, number of events; PsA, psoriatic arthritis; PsO, psoriasis; TEAE, treatment-emergent adverse event.

**Table S5:** IRs per 100 PY for malignancies across indications, over the time periods examined.

|  | **Pooled PsO IXE**  **(N=6892)** | **Pooled PsA IXE**  **(N=1401)** | **Pooled axSpA IXE**  **(N=932)** |
| --- | --- | --- | --- |
|  | Total PY: 18025.7  IR per 100 PY (95%CI) | Total PY: 2247.7  IR per 100 PY (95%CI) | Total PY: 2097.7  IR per 100 PY (95%CI) |
| Patients with >=1 TEAE Malignancies | 0.8 (0.7, 0.9) | 0.7 (0.4,1.1) | 0.4 (0.2, 0.8) |
| NMSC  BCC  SCC | 0.3 (0.2, 0.4)  0.2 (0.2, 0.3)  0.1 (0.1, 0.1) | 0.4 (0.2, 0.8)  0.3 (0.1, 0.6)  0.2 (0.1, 0.5) | 0 (0.0, 0.0)  -  - |
| Malignancies excluding NMSC | 0.5 (0.4, 0.6) | 0.3 (0.1, 0.7) | 0.4 (0.2, 0.8) |

axSpA, axial spondyloarthritis; BCC, Basal Cell Carcinoma; CI, confidence interval; IR, Incidence rate; IXE,

Ixekizumab; N, number of patients in the analysis population; n, number of patients with at least one

treatment-emergent malignancy event; NMSC, Non-Melanoma Skin Cancer; PsA, psoriatic arthritis; PsO,

psoriasis; PY, patient-years; SCC, Squamous Cell Carcinoma.

**Table S6:** Selected AEs of interest examined by sex

|  | **PsO IXE**  **(N=6892)** | | | | | | **PsA IXE**  **(N=1401)** | | | | | | **axSpA**  **(N=932)** | | | | | |
| --- | --- | --- | --- | --- | --- | --- | --- | --- | --- | --- | --- | --- | --- | --- | --- | --- | --- | --- |
|  | **Female** | | | **Male** | | | **Female** | | | **Male** | | | **Female** | | | **Male** | | |
|  | n (%) | IR | CI | n (%) | IR | CI | n (%) | IR | CI | n (%) | IR | CI | n (%) | IR | CI | n (%) | IR | CI |
| TEAE  Mild  Moderate  Severe | 1998 (86.5)  572 (24.8)  1070 (46.3)  356 (15.4) | 34.6  9.9  18.5  6.2 | 33.1, 36.1  9.1, 10.7  17.4, 19.7  5.6, 6.8 | 4031 (84.4)  1308 (27.4)  2034 (42.6)  688 (14.4) | 32.0  10.4  16.2  5.5 | 31.0, 33.0  9.8, 11.0  15.5, 16.9  5.1, 5.9 | 597 (82.7)  235 (32.5)  300 (41.6)  62 (8.6) | 52.3  20.6  26.3  5.4 | 48.2, 56.6  18.1, 23.4  23.5, 29.4  4.2, 7.0 | 534 (78.6)  226 (33.3)  256 (37.7)  52 (7.7) | 48.3  20.4  23.2  4.7 | 44.4, 52.6  17.9, 23.3  20.5, 26.2  3.6, 6.2 | 254 (90.1)  79 (28.0)  139 (49.3)  36 (12.8) | 42.8  13.3  23.4  6.1 | 37.9, 48.5  10.7, 16.6  19.9, 27.7  4.4, 8.4 | 544 (83.7)  197 (30.3)  280 (43.1)  67 (10.3) | 36.1  13.1  18.6  4.5 | 33.2, 39.3  11.4, 15.1  16.5, 20.9  3.5, 5.7 |
| Death | 8 (0.3) | 0.1 | 0.1, 0.3 | 28 (0.6) | 0.2 | 0.2, 0.3 | 2 (0.3) | 0.2 | 0.0, 0.7 | 4 (0.6) | 0.4 | 0.1, 1.0 | 0 | 0.0 | 0.0, 1.3 | 3 (0.5) | 0.2 | 0.1, 0.6 |
| Serious AE | 331 (14.3) | 5.7 | 5.1, 6.4 | 653 (13.7) | 5.2 | 4.8, 5.6 | 73 (10.1) | 6.4 | 5.1, 8.0 | 61 (9.0) | 5.5 | 4.3, 7.1 | 33 (11.7) | 5.6 | 4.0, 7.8 | 68 (10.5) | 4.5 | 3.6, 5.7 |
| Discontinuation due to AE (including death) | 205 (8.9) | 3.5 | 3.1, 4.1 | 319 (6.7) | 2.5 | 2.3, 2.8 | 68 (9.4) | 6.0 | 4.7, 7.6 | 47 (6.9) | 4.3 | 3.2, 5.7 | 26 (9.2) | 4.4 | 3.0, 6.4 | 40 (6.2) | 2.7 | 1.9, 3.6 |
| **Selected AEs of interest**  Infection  Allergic reactions  Injection-site reactions  Hepatic  Cerebro-cardiovascular events  Depression  Cytopenias  MACE  Malignancies  IBD | 1531 (66.2)  383 (16.6)  478 (20.7)  117 (5.1)  42 (2.0)  98 (4.2)  51 (2.2)  19 (0.9)  49 (2.1)  13 (0.6) | 26.5  6.6  8.3  2.0  0.8  1.7  0.9  0.4  0.8  0.2 | 25.2, 27.9  6.0, 7.3  7.6, 9.0  1.7, 2.4  0.6, 1.1  1.4, 2.1  0.7, 1.2  0.2, 0.6  0.6, 1.1  0.1, 0.4 | 2921 (61.1)  651 (13.6)  618 (12.9)  391 (8.2)  144 (3.2)  125 (2.6)  123 (2.6)  72 (1.6)  93 (1.9)  17 (0.4) | 23.2  5.2  4.9  3.1  1.2  1.0  1.0  0.6  0.7  0.1 | 22.4, 24.1  4.8, 5.6  4.5, 5.3  2.8, 3.4  1.0, 1.4  0.8, 1.2  0.8, 1.2  0.5, 0.7  0.6, 0.9  0.1, 0.2 | 413 (57.2)  63 (8.7)  177 (24.5)  44 (6.1)  12 (1.7)  22 (3.0)  28 (3.9)  4 (0.6)  8 (1.1)  2 (0.3) | 36.2  5.5  15.5  3.9  1.1  1.9  2.5  0.4  0.7  0.2 | 32.8, 39.8  4.3, 7.1  13.4, 18.0  2.9, 5.2  0.6, 1.8  1.3, 2.9  1.7, 3.6  0.1, 0.9  0.4, 1.4  0.0, 0.7 | 346 (51.0)  39 (5.7)  83 (12.2)  68 (10.0)  16 (2.4)  15 (2.2)  28 (4.1)  8 (1.2)  7 (1.0)  1 (0.1) | 31.3  3.5  7.5  6.2  1.4  1.4  2.5  0.7  0.6  0.1 | 28.2, 34.8  2.6, 4.8  6.1, 9.3  4.9, 7.8  0.9, 2.4  0.8, 2.3  1.7, 3.7  0.4, 1.4  0.3, 1.3  0.0, 0.6 | 190 (67.4)  25 (8.9)  65 (23.0)  20 (7.1)  3 (1.1)  8 (2.8)  5 (1.8)  0  6 (2.1)  8 (2.8) | 32.0  4.2  11.0  3.4  0.5  1.3  0.8  0.0  1.0  1.3 | 27.8, 36.9  2.8, 6.2  8.6, 14.0  2.2, 5.2  0.2, 1.6  0.7, 2.7  0.4, 2.0  0.0, 1.3  0.5, 2.3  0.7, 2.7 | 350 (53.8)  63 (9.7)  91 (14.0)  60 (9.2)  15 (2.3)  11 (1.7)  23 (3.5)  6 (0.9)  3 (0.5)  9 (1.4) | 23.3  4.2  6.0  4.0  1.0  0.7  1.5  0.4  0.2  0.6 | 20.9, 25.8  3.3, 5.4  4.9, 7.4  3.1, 5.1  0.6, 1.7  0.4, 1.3  1.0, 2.3  0.2, 0.9  0.1, 0.6  0.3, 1.1 |

CI: 95% CI of IR. TEAE by severity: Patients with multiple occurrences of the same event are counted under the highest severity. Serious AE: The data collection for the clinical trial database does not contain specification on when events become serious; the numbers may represent more events considered serious than what was actually serious during the treatment period. Cerebro-cardiovascular and MACE events were all confirmed cases. IBD cases were confirmed by adjudication. Cytopenias were based on SMQ. Percentage was calculated by n/N*100%. CIs of IR are from the likelihood ratio test of treatment effect from the Poisson regression. AE, adverse event; axSpA, axial spondyloarthritis; CI, confidence interval; IBD, inflammatory bowel disease; IR, incidence rate; IXE, ixekizumab; MACE, major adverse cerebro-cardiovascular event; N, number of patients in the analysis population; n, number of patients in each category; PsA, psoriatic arthritis; PsO, psoriasis; SMQ, standardized MedDRA queries; TEAE, treatment-emergent adverse event.
