# Supplementary figures and images for: Long-term safety of Ixekizumab in adults with psoriasis, psoriatic arthritis, or axial spondyloarthritis: a post-hoc analysis of final safety data from 25 randomized clinical trials
Source: Arthritis Res Ther. 2024 Feb 12;26:49. doi: 10.1186/s13075-023-03257-7 (PMC10860236; doi:10.1186/s13075-023-03257-7)

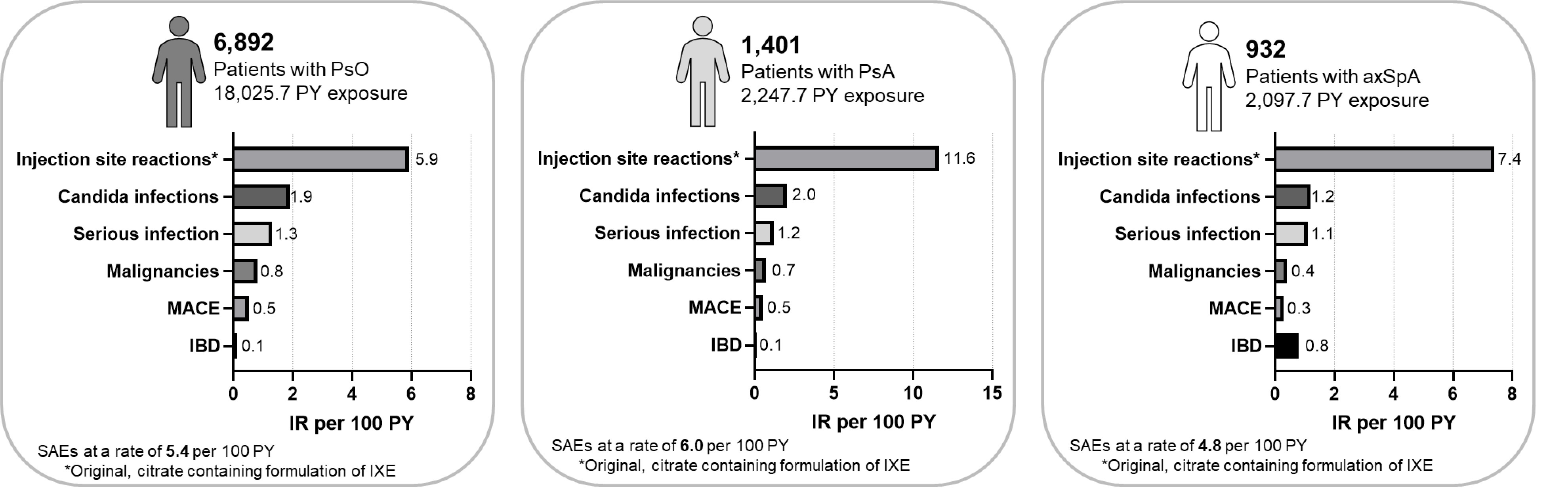

Supplement: Supplementary file 2 — Additional file 2. Study Infographic. Overview of Safety Outcomes across Indications. [file 13075_2023_3257_MOESM2_ESM.jpg]
